# Use of electronic health record data mining for heart failure subtyping

**DOI:** 10.1186/s13104-023-06469-x

**Published:** 2023-09-11

**Authors:** Matti A. Vuori, Tuomo Kiiskinen, Niina Pitkänen, Samu Kurki, Hannele Laivuori, Tarja Laitinen, Sampo Mäntylahti, Aarno Palotie, FinnGen, Teemu J. Niiranen

**Affiliations:** 1 Division of Medicine, University of Turku, Kiinamyllynkatu 10, Turku, FI-20520 Finland; 2 Turku University Hospital, Kiinamyllynkatu 4-8, Box 52, Turku, FI-20521 Finland; 3 Institute for Molecular Medicine Finland (FIMM), HiLIFE, University of Helsinki, Tukholmankatu 8, Helsinki, Finland; 4 Auria Biobank, Kiinamyllynkatu 10, PO Box 30, Turku, FI-20520 Finland; 5 Centre for Child, Adolescent, and Maternal Health Research, Faculty of Medicine and Health Technology, Tampere University, Tampere, Finland; 6 Department of Obstetrics and Gynecology, Tampere University Hospital, Tampere, Finland; 7 Administration Center, Tampere University Hospital and University of Tampere, P.O. Box 2000, Tampere, 33521 Finland; 8 Helsinki Biobank, Haartmaninkatu 3, Helsinki, 00290 Finland; 9 Department of Public Health Solutions, Finnish Institute for Health and Welfare, PO Box 30, Helsinki, FI-00271 Finland

**Keywords:** Heart failure, Ejection fraction, Data mining, Text mining, Electronic health records, HFrEF, HFpEF, HFmrEF

## Abstract

**Objective:**

To assess whether electronic health record (EHR) data text mining can be used to improve register-based heart failure (HF) subtyping. EHR data of 43,405 individuals from two Finnish hospital biobanks were mined for unstructured text mentions of ejection fraction (EF) and validated against clinical assessment in two sets of 100 randomly selected individuals. Structured laboratory data was then incorporated for a categorization by HF subtype (HF with mildly reduced EF, HFmrEF; HF with preserved EF, HFpEF; HF with reduced EF, HFrEF; and no HF).

**Results:**

In 86% of the cases, the algorithm-identified EF belonged to the correct HF subtype range. Sensitivity, specificity, PPV and NPV of the algorithm were 94–100% for HFrEF, 85–100% for HFmrEF, and 96%, 67%, 53% and 98% for HFpEF. Survival analyses using the traditional diagnosis of HF were in concordance with the algorithm-based ones. Compared to healthy individuals, mortality increased from HFmrEF (hazard ratio [HR], 1.91; 95% confidence interval [CI], 1.24–2.95) to HFpEF (2.28; 1.80–2.88) to HFrEF group (2.63; 1.97–3.50) over a follow-up of 1.5 years. We conclude that quantitative EF data can be efficiently extracted from EHRs and used with laboratory data to subtype HF with reasonable accuracy, especially for HFrEF.

**Supplementary Information:**

The online version contains supplementary material available at 10.1186/s13104-023-06469-x.

## Introduction

Heart failure (HF) is an end-stage cardiac condition in which the heart pumping function is insufficient. HF has been a challenging outcome in epidemiological studies as its subtypes are often impossible to discern using solely register data [1]. In many countries, the International Classification for Diseases (ICD) holds only a single diagnostic code for congestive HF, and has no separate codes for HF with reduced ejection fraction (EF; HFrEF), HF with mildly reduced EF (HFmrEF), or HF with preserved EF (HFpEF) [2–4].

In this study, we set out to combine register data from the FinnGen database with information mined from electronic health records (EHR) to improve subtyping of register-based HF diagnoses [5]. We assessed the feasibility of EHR mining for non-structured text mentions for EF values, and whether EHR-mined HF subtypes could be used effectively to discern mortality risk. Information gained from this study could be used to determine HF subtypes to be further used in future research purposes of the very heterogeneous HF syndrome.

## Materials and methods

### Study sample

FinnGen is a joint research project aiming to collect the genomic and EHR data of 500,000 Finns from population-based studies and hospital biobanks [5]. The FinnGen register database holds individual-level health information mainly based on ICD-10 coding from nationwide registers, such as the Finnish Hospital Discharge Register (since 1968) and the Causes of Death Register (since 1969). These data enable defining a large number of clinical end points, including HF [4, 6]. The registers do not contain any EHR data.

FinnGen participants’ data in the Auria (Turku, Finland; n = 29,201) and Helsinki (Helsinki, Finland; n = 58,693) hospital biobanks were accessed for this study, with data collected in 2001–2020. Our data mining algorithm identified EF data for 43,405 individuals. Data was available for 35,800 individuals after excluding individuals with missing creatinine (available for n = 40,864) and N-terminal-pro-b-type natriuretic peptide (proBNP, available for n = 9479) laboratory parameters. ProBNP was only required for HF cases. After removal of fatal cases with missing baseline HF information (n = 534) or missing HF follow-up data (n = 1,283), our study sample consisted of 33,983 participants.

### EHR data mining algorithm

To study whether HF subtyping based on EHRs is possible and feasible, we created a rule-based, regular expressions, and string-matching algorithm for data mining purposes. First, the EHR and clinical reports were text mined for all references to EF. Second, proBNP and creatinine were drawn from structured laboratory data. The main EHR data were then merged with the register-based FinnGen clinical data using personal identification codes that are unique for each Finnish resident.

**Fig. 1:**
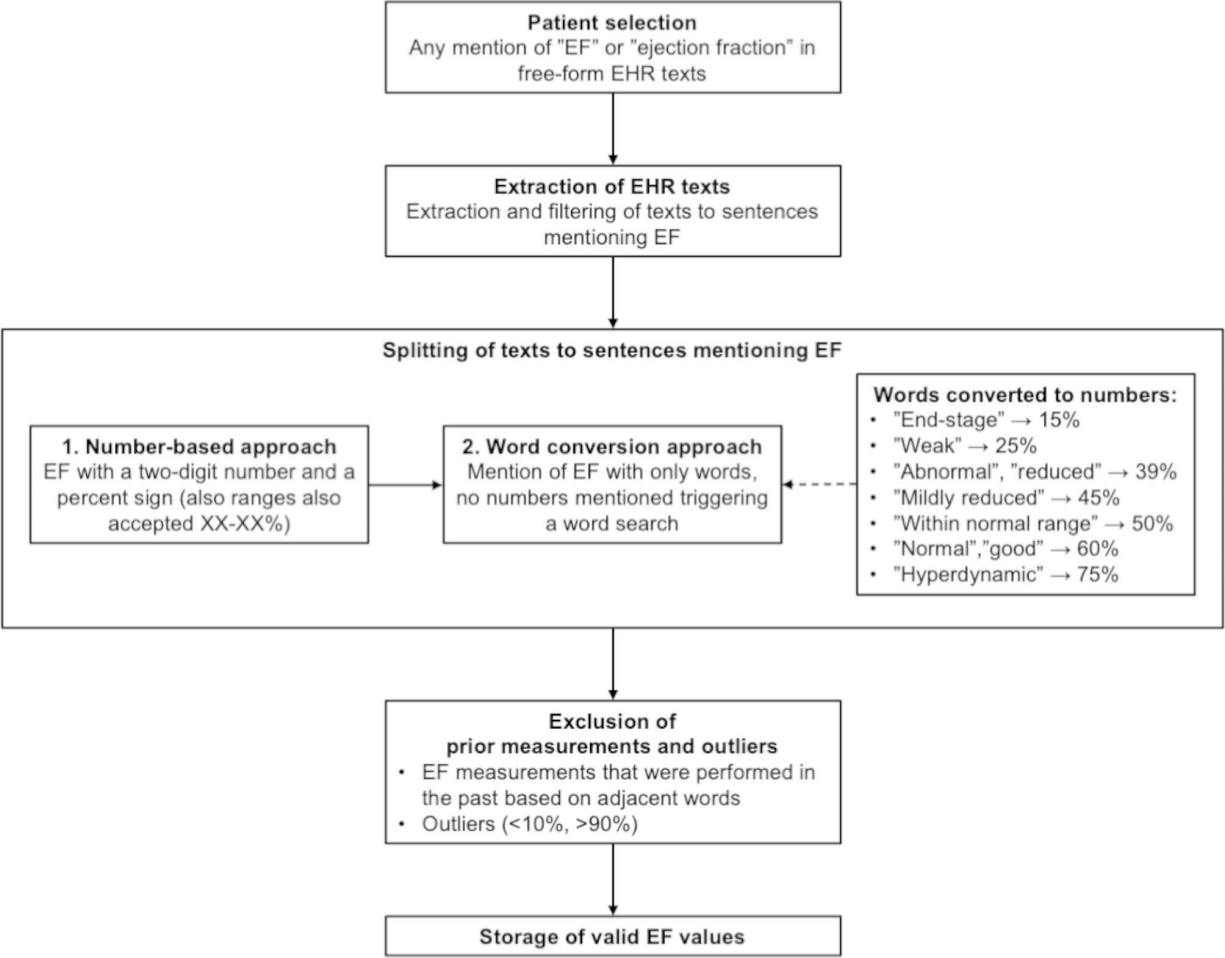
The principle of the EF mining algorithm Abbreviations: EF, ejection fraction; EHR, electronic health records; HFrEF, heart failure with reduced ejection fraction (< 40%); HFmrEF, heart failure with mildly reduced ejection fraction (40–49%); HFpEF, heart failure with preserved ejection fraction (≥ 50%)

The overarching principles of the algorithm are presented in Fig. 1. First, the algorithm searches for mentions of “EF” or “ejection fraction” from the EHRs. When these terms are observed, the texts are extracted, filtered, and split to sentences and the sentences are searched first for a series of two numbers that could be an EF measurement; two digits after each other and a percent marker, or the word ‘percent’. Ranges are also searched with two series of two digits and a percent marker, separated by a hyphen. Clinicians also use a wide variety of expressions for describing EF. If no numbers are present, a word search is triggered. The words describing EFaere converted to numbers based on the 2016 European Society of Cardiology (ESC) HF guidelines [2]. I.e., we defined “preserved”, “mildly reduced”, and “reduced” ejection fraction as 50%, 45%, and 39% to meet with the ESC definitions. The definitions for the other common worded descriptions of EF were defined based on clinical judgment. In addition, all sentences undergo a simultaneous quality check to exclude dates possibly masquerading as EF readings, and EF readings done in the past (e.g., “EF 40% a year ago” is disqualified). A mean EF is calculated if several EF readings are observed at the same date. EF outliers (< 10% or > 90%) are also removed. The code for the algorithm is available online at: https://zenodo.org/record/7900516#.ZFi92S9Z9qs.

### HF subgrouping

Based on the mined EF and proBNP values, the participants were categorized into four clinical HF subtypes based on the ESC guideline [2] by the algorithm: (1) no HF was defined as normal EF (here defined as ≥ 50%) and normal proBNP levels (≤ 125 ng/ml); (2) HFrEF was defined as EF < 40%; (3) HFmrEF was defined as EF 40–49%; (4) HFpEF was defined as EF ≥ 50% and proBNP levels of ≥ 125 ng/ml.

### Validation procedures

After data extraction, two validations with separate 100 randomly selected individuals were undertaken. First, we examined all the mined instances of EF values for the first 100 individuals and an internist (M.V.) defined a correct EF value for a specific time-point from the EHR data without knowing the algorithm-defined EF. The algorithm-defined EF values were measured against the gold-standard clinician-defined values. Subsequently, the HF subtype was defined for another 100 patients (with also proBNP values available) by the algorithm and by the internist blinded from the results of the algorithm. The misclassified cases were reviewed and the reasons for an inaccurate EF reading and subtyping were identified.

### Statistical analyses

To test the functionality of the algorithm, Cox proportional hazards models were used to assess the association between HF subtypes with overall mortality, with individuals with no HF as the reference. We adjusted for risk factors that are common in HF and also increase the risk of death – sex, estimated glomerular filtration rate [7], and register-based diagnoses of prevalent hypertension, ischemic heart disease, type 2 diabetes, chronic obstructive pulmonary disease, and renal failure. Age was used as the time scale. The definitions of comorbidities in FinnGen are available online at https://risteys.finngen.fi. Proportional hazards assumptions were assessed by inspecting visually plotted Schoenfeld residuals.

## Results

### Study sample and data mining results

The characteristics of the study sample are presented in Table 1. A slight majority of the sample were women (58.1%), and the mean age was 58.7 (standard deviation 18.2). The most common clinical comorbidities were hypertension (29.7%), type 2 diabetes mellitus (17.2%) and coronary artery disease (15.7%). After dividing the participants into subphenotypes according to the algorithm, 1,162 had HFrEF, 474 had HFmrEF, 2,110 had HFpEF, and 30,237 had no HF.

**Table 1:** Study sample characteristics

Characteristic	Whole sample	HFrEF	HFpEF	HFmrEF	No HF
N	33,983 (100)	1,162 (3.4)	2,110 (6.2)	474 (1.4)	30,237 (89.0)
Women	19,735 (58.1)	465 (40.0)	1,056 (50.0)	137 (28.9)	18,077 (59.8)
Age, years, mean (SD)	58.7 (18.2)	67.9 (14.6)	70.8 (14.6)	68.8 (13.1)	57.3 (18.1)
Medical History					
Coronary artery disease	5,325 (15.7)	510 (43.9)	886 (42.0)	260 (54.9)	3,669 (12.1)
AF	4,676 (13.8)	538 (46.3)	1,043 (49.4)	271 57.2)	2,824 (9.3)
Hypertension	10,078 (29.7)	684 (58.9)	1,506 (71.4)	293 (61.8)	7,595 (25.1)
Cardiomyopathy	874 (2.6)	336 (28.9)	152 (7.2)	115 (24.2)	272 (0.9)
Type 2 diabetes mellitus	5,836 (17.2)	440 (37.9)	754 (35.7)	165 (34.8)	4,477 (14.8)
Renal insufficiency	1,103 (3.2)	175 (15.1)	425 (20.1)	68 (14.3)	435 (1.4)
COPD	1,373 (4.0)	139 (12.0)	261 (12.4)	64 (13.5)	909 (3.0)
proBNP, ng/l, mean (SD)	1,666 (4,587)	3,087 (6,877)	2,357 (6,000)	3,259 (6,318)	77 (25)
eGFR, ml/min/1.73 m^2^, mean (SD)	91 (27)	84 (26)	81 (27)	84 (23)	97 (27)
Mined EF, %, mean (SD)	49.0 (10.3)	34.2 (6.5)	60.8 (7.7)	43.8 (2.9)	63.0 (8.4)

Numbers are presented as n (%) unless otherwise indicated

Abbreviations: EF, ejection fraction; HFrEF, heart failure with reduced ejection fraction (< 40%); HFmrEF, heart failure with mildly reduced ejection fraction (40–49%); HFpEF, heart failure with preserved ejection fraction ($\ge$50%); AF, atrial fibrillation; COPD, chronic obstructive pulmonary disease; proBNP, N-terminal pro b-type natriuretic peptide; eGFR, estimated glomerular filtration rate

### Validation

The assessment of the clinician and the algorithm resulted in the same EF in 78% of the patients. In 87% of patients, the algorithm-mined EF value was within a 5% range with the clinician’s estimate, and in 86% of patients, the algorithm-derived EF value was in the correct HF subtype range. In the 22 cases where the algorithm missed the right EF, the reasons were the inability to find the correct EF value (12 cases) and the calculation of mean EF from an incorrect and correct EF value (10 cases). Results and metrics of the HF subtype validation are presented in Table 2. The performance of the algorithm was good in detecting HF in general. However, false positives, all due to proBNP being elevated for a reason other than HF limited the performance of the algorithm for diagnosing HFpEF.

**Table 2:** Results of the HF subtype validation and calculated epidemiological measures

Category	HF based on clinician’s diagnosis					Calculated epidemiological measures				
Algorithm-based HF	HF, all	HFrEF	HFmrEF	HFpEF	No HF	Sensitivity	Specificity	PPV	NPV	Accuracy
HF, all	63	18	17	28	25	100%	32%	72%	100%	75%
HFrEF	17	17	0	0	0	94%	100%	100%	99%	99%
HFmrEF	19	1	17	1	1	100%	96%	85%	100%	97%
HFpEF	27	0	0	27	24	96%	67%	53%	98%	75%
No HF	12	0	0	0	12	32%	100%	100%	72%	75%

The algorithm defined ‘No HF’ as EF ≥ 50% and proBNP ≤ 125 ng/ml, ‘HF’ as any HF subtype present, ‘HFrEF’ as EF < 40%, ‘HFmrEF’ as EF 40–49% and ‘HFpEF’ as EF ≥ 50% and proBNP ≥ 125 ng/ml, using EF values mined by the algorithm. The clinician made the diagnosis of HF subtype based on EHR text, EF report and proBNP values

Abbreviations: EF, ejection fraction; HF, heart failure; HFrEF, HF with reduced EF; HFmrEF, HF with mildly reduced EF; HFpEF, HF with preserved EF; PPV, positive predictive value; NPV, negative predictive value; proBNP, N-terminal pro b-type natriuretic peptide

### Risk of death by EF subtype

The multivariate-adjusted risk of death for a register-based diagnosis of HF, as compared to individuals with no HF, was 2.35-fold (95% CI, 1.90–2.90). For an algorithm-based diagnosis of HF (any subtype), this risk was 2.47-fold (95% CI, 2.00–3.06) (Supplementary Table 1). When analyzing the risk of death for algorithm-based subtypes, the highest HR was observed for HFrEF, 2.63 (95% CI, 1.97–3.50), as expected. The risks of death in the HFmrEF and HFpEF groups were 1.91-fold (95% CI, 1.24–2.95) and 2.28-fold (95% CI, 1.80–2.88), as compared to individuals with no HF according to the algorithm. In the study sample, 3,875 individuals had the gold standard EHR-based diagnosis of HF, in comparison to 3,746, when using the algorithm to define HF. The mean follow-up time was 1.5 (SD 1.2) years.

## Discussion

In this study, we generated a data mining algorithm for extracting free-text EF values and laboratory data for improving HF subclassification.

Although the EF provided by the algorithm had 78–86% concordance with clinical assessment, EF was a challenging target for text mining. The greatest challenge for the algorithm was to correctly distinguish the current EF value from previous EF measurements that were often listed in the same unstructured text. However, this limitation was overcome surprisingly well by using mean EF values. The word search and numeric conversion functioned well in general, and descriptive reports did not tend be a problem. The mining of laboratory values was unproblematic as it was always based on structured data.

The risk of death was similar in both mortality analyses, and significantly lower in the group with HFmrEF compared to those with HFrEF or HFpEF. This finding is in line with a meta-analysis of 12 observational studies with 109,257 HF patients by Lauritsen et al. [8]. The profile of comorbid conditions in our study sample was also similar to that of the meta-analysis. The agreement between our findings from our study and the study by Lauritsen et al. provide further support on the validity of our data mining algorithm.

To our knowledge, text mining of EF values has not been attempted previously. In contrast, text mining of several dichotomous disease states has been previously attempted, such as for pregnancy status in a sample of 344 patients [9], the presence of colorectal cancer in a sample of 1,262,671 patient reports and pathology notes [10], systematic lupus erythematosus (SLE) in a sample of 4,607 patients [11], and cardiac implantable device infections in a sample of 19,212 implant procedure patients records [12]. In these studies, Labrosse [9], Brunekreef [11] and Mull [12] used a string character or rule-based text mining algorithm that was very similar to ours and resulted in analogous results. The accuracy of SLE detection was very similar to ours: 71% had a complete agreement in diagnosis in a validation sample of 100 randomly selected patients [11]. Labrosse et al. [9] manually reviewed all records, and their algorithm was superior to detecting pregnancy (35 of 36) compared to manual EHR assessment (30 of 36). Mull et al. reviewed 232 records of patients with a high risk of implantable device infection [12]. Text mining yielded a low positive predictive value (PPV) of 43.5% for the algorithm, but a very good sensitivity 94.4%, like in our study for HFpEF. Finally, Xu et al. manually validated a set of 300 patient records for the presence of colorectal cancer [10]. In this study, natural language processing provided a PPV of 84%. The main limiting factor in these studies is the relatively high number of false positives resulting in low PPVs. As the idea of the algorithm is to read through large volumes of patient data, high accuracy is needed for the mined data to be useful in clinical practice or research.

We conclude that quantitative EF and laboratory data can be efficiently extracted from EHRs and that these data can be used to subtype HF with reasonable accuracy, especially for HFrEF. The better and more clearly-defined the algorithm-defined subtypes are, the more the results of the future studies using definitions of HF subtypes derived from these will be expected to be concise.

### Limitations

Our study has certain limitations. The algorithm performed well in capturing HFrEF and HFmrEF subtypes, but proBNP values elevated for a reason other than HF made it less capable in diagnosing HFpEF. Although the algorithm classified 86% of HF patients under the correct HF subtype, the accuracy of the mined EF values needs to be further improved. Particularly HFpEF detection could be improved by implementing concurrent comorbidity information to better discern the reasons for proBNP elevation. Also, echocardiographic markers of diastolic dysfunction could further improve HFpEF diagnosis. Unfortunately, these markers were usually not recorded in most clinical echocardiography reports until very recently, rendering this approach impossible for now. In addition, individual HF timelines with longitudinal information on the disease pattern could be incorporated, aiming to discern various chronic HF subtypes. Finally, machine learning approaches such as natural language processing could possibly lead to improved language processing.

Furthermore, the validations were performed by a single blinded clinician who reviewed only two sets of 100 cases.

### Electronic supplementary material

Below is the link to the electronic supplementary material.

Supplementary Material 1

Supplementary Material 2

## Data Availability

The FinnGen data may be accessed through Finnish Biobanks’ FinBB portal (web link: www.finbb.fi, email: info.fingenious@finbb.fi).

## List of abbreviations

CI: Confidence interval
COPD: Chronic obstructive pulmonary disease
EF: Ejection fraction
eGFR: Estimated glomerular filtration rate
EHR: Electronic health records
ESC: The European Society of Cardiology
HF: Heart failure
HFmrEF: Heart failure with mildly reduced ejection fraction
HFpEF: Heart failure with preserved ejection fraction
HFrEF: Heart failure with reduced ejection fraction
HR: Hazard ratio
ICD: International Classification for Diseases
NPV: Negative predictive value
PPV: Positive predictive value
proBNP: N-terminal-pro-b-type natriuretic peptide
