# Supplementary material for: Use of electronic health record data mining for heart failure subtyping
Source: BMC Res Notes. 2023 Sep 11;16:208. doi: 10.1186/s13104-023-06469-x (PMC10496250; doi:10.1186/s13104-023-06469-x)
Supplement: Supplementary file 1 — Supplementary Material 1 [file 13104_2023_6469_MOESM1_ESM.docx]

**Additional File 1**

**Supplementary Table 1.** Risk of death by HF subtype in two separate mortality analyses, using the EHR-defined HF endpoint, and using the algorithm-defined HF and its subtypes.

| Category | Definition | *n* | *n* of deaths | Hazard ratio (95% CI) | |
| --- | --- | --- | --- | --- | --- |
| HF based on EHR diagnosis |  | 33983 | 659 | |  |
| No HF | No HF diagnosis present in the EHRs | 30108 | 445 | | 1.00 (reference) |
| HF | HF diagnosis present in the EHRs | 3875 | 214 | | 2.35 (1.90–2.90) |
| Algorithm-based HF |  | 33983 | 659 | |  |
| No HF | EF (algorithm) ≥ 50 % and proBNP ≤125 ng/ml | 30237 | 446 | | 1.00 (reference) |
| HF | Any HF subtype present (algorithm) | 3746 | 213 | | 2.47 (2.00–3.06) |
| HF with reduced EF | EF (algorithm) < 40 % | 1162 | 70 | | 2.63 (1.97–3.50) |
| HF with mildly reduced EF | EF (algorithm) 40–49 % | 474 | 24 | | 1.91 (1.24–2.95) |
| HF with preserved EF | EF (algorithm) ≥ 50 % and proBNP ≥ 125 ng/ml | 2110 | 119 | | 2.28 (1.80–2.88) |

Hazard ratios are adjusted for age, sex, estimated glomerular filtration rate, and the following baseline diseases: coronary artery disease, atrial fibrillation, hypertension, cardiomyopathy, type 2 diabetes mellitus, renal insufficiency, and chronic obstructive pulmonary disease. Abbreviations: EF, ejection fraction; HF, heart failure; proBNP, N-terminal pro b-type natriuretic peptide; CI, confidence interval.
